# Nitrous oxide emission factors from fertilizer use in Brazilian agricultural systems: meta-analytical insights for soil management, climate, and land-use policies

**DOI:** 10.1007/s10661-026-15560-2

**Published:** 2026-06-26

**Authors:** Sandra Furlan Nogueira, Nilza Patrícia Ramos, Joaquim Ernesto Bernardes Ayer, Cristiano Alberto de Andrade, Ricardo Antonio Almeida Pazianotto, Marcos Antônio Ligo, Danilo Francisco Trovo Garofalo, Isa Rolisola, Ana Paula Contador Packer, Marilia Ieda da Silveira Folegatti

**Affiliations:** Embrapa Environment, SP-340 Highway, km 127.5, s/n, Tanquinho Velho, Jaguariúna, SP 13918-110 Brazil

**Keywords:** Climate change mitigation, Agricultural management, Grain crops, Pasture systems, Sugarcane production

## Abstract

Mineral fertilizers account for 18% of Brazil’s agricultural greenhouse gas (GHG) emissions, as reported in the Fourth National Inventory. Initial estimates relied on IPCC Tier I factors, but recent methodologies have advanced toward Tier II and III approaches, incorporating fertilizer type and regional conditions. This study aimed to refine direct N₂O emission estimates by proposing specific emission factors (EFs) for organic, mineral, and combined fertilizers across sugarcane, grain, and pasture systems. These factors considered environmental conditions, agricultural practices, and excreta inputs to improve national representativeness. A systematic review of 56 articles from Web of Science, Scopus, and SciELO yielded 325 trials. Variables analyzed included climate, biome, soil type, irrigation, management, nitrogen sources and rates, pH, texture, and soil carbon, nitrogen, and organic matter. Statistical analyses included boxplots, Kruskal–Wallis tests, and meta-analysis with bootstrapping, all performed in R. Higher N₂O emission factors were generally observed in pasture systems under Cwa climatic conditions, in grain systems under subtropical climates (Cfb and Cfa), and in sugarcane systems under tropical and subtropical climates (Am and Cfa), with elevated values frequently observed in the Cerrado and Atlantic Forest biomes. Weighted EFs ranged from 0.14 to 1.63%, while national average EFs were 0.66 for sugarcane, 0.69 for grains, and 0.64 for pastures, all within the uncertainty range of IPCC default values. These results demonstrate the potential of Tier II factors to improve N₂O emission estimates in Brazil, while highlighting important limitations related to data availability, methodological heterogeneity, and uneven geographic coverage. Continued efforts to expand regionally representative datasets and advance toward Tier III approaches are essential to reduce uncertainties and support more robust GHG inventories, life cycle assessments, and mitigation strategies.

**Graphical abstract:**

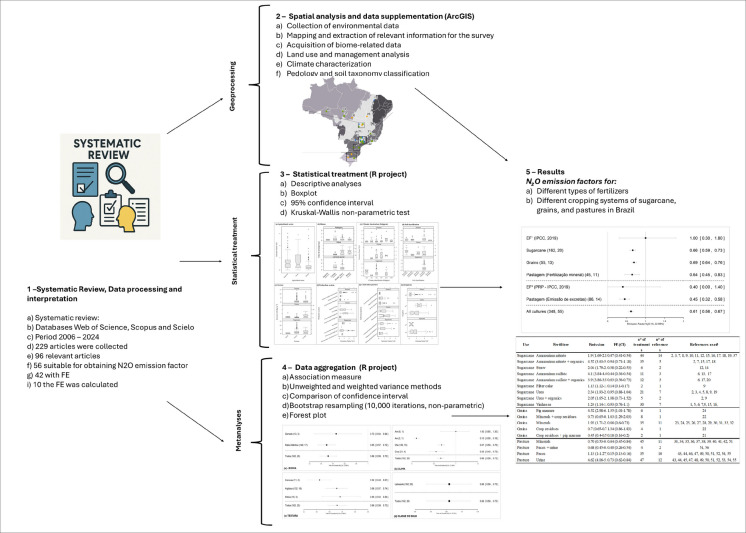

**Supplementary Information:**

The online version contains supplementary material available at 10.1007/s10661-026-15560-2.

##  Introduction

Over the past 30 years (1993–2023), Brazil expanded its planted agricultural and pasture areas by 115% and 27%, respectively. The total area of temporary crops grew from 29 to 59 Mha, while cultivated pasture increased from 130 to 164 Mha. Soybean acreage tripled, and sugarcane areas quadrupled during this period (MapBiomas, 2025). Corn cultivation followed a different pattern: first-season corn areas declined by 60%, from 11 to 4.4 Mha, while second-crop corn areas increased more than 12-fold, from 1.3 Mha to 16.4 Mha (CONAB, 2025).

Land use conversion in Brazil over the past two decades has been highly dynamic, driven primarily by the replacement of pasture areas with soybean (Garofalo et al., 2022; MapBiomas, 2025; Song et al., 2021) and sugarcane crops (Adami et al., 2012; Garofalo et al., 2022; MapBiomas, 2025). Despite this agricultural expansion, pasture areas have remained relatively stable at around 160 Mha, largely due to the growth of pastures in the Amazon biome (MapBiomas, 2025). Moreover, productivity has increased during this period, mainly as a result of livestock intensification (ABIEC, 2021).

Thus, land conversion into productive environments has occurred and continues to take place, both along agricultural frontiers through deforestation, and more commonly through the transformation of degraded agricultural areas. These areas, typically occupied by extensive pasture, are increasingly being converted into croplands, primarily for corn, soybean, sugarcane, and cotton production (Ayer et al., 2019, 2021; MapBiomas, 2025). This process has been supported by techniques for correcting soil acidity and the application of mineral and organic fertilizers since Brazilian soils are naturally highly weathered, acidic, low in P and K, and have limited N availability (Ayer et al., 2024; Bertol, et al., 2019).

The expansion of Brazilian agribusiness, coupled with rising fertilizer use, has positioned the country as the world’s fourth-largest consumer of these inputs. Between 2000 and 2020, fertilizer consumption grew by 145%, from 16.4 to 40 million tons. Soybeans, corn, sugarcane, cotton, and coffee together account for over 80% of national fertilizer demand. The amount of nitrogen supplied to producers increased by 86% between 2010 and 2018. In 2018, Brazil imported approximately 9 million tons of nitrogen fertilizers, twice the volume imported in 2008. Of this total, 61% was urea, 25% ammonium sulfate, 11% ammonium nitrate, and 3% ammonia (Anda, 2021).

In 2019, global agricultural emissions, including those from agricultural practices and land-use change, reached nearly 11 billion tons of CO₂ equivalent (CO₂ eq). Brazil, Indonesia, and China together accounted for more than 50% of these emissions. Non-CO₂ gases (methane and nitrous oxide), along with CO₂ emissions from agricultural activities, contributed over 7 billion tons of CO₂ eq in 2019, representing a 9% increase since 1990. Approximately two-thirds of non-CO₂ emissions were linked to livestock production (FAO, 2021).

According to the Fourth National Inventory (Brazil, 2020), N₂O emissions from the agricultural sector totaled 510.46 Gg in 2016, accounting for 87.1% of the country’s emissions of this gas. Within this sector, direct emissions from soil management represented 74.4%, while indirect emissions contributed 22.3%. Emissions from animal waste management and the burning of agricultural residues accounted for 3.1% and 0.2%, respectively. Mineral fertilizers alone contributed 18% of agricultural GHG emissions, equivalent to 2.5% of total anthropogenic emissions.

To estimate direct N₂O emissions, the initial national inventories applied Tier I equations and calculation factors (IPCC, 2019), where emission patterns are expressed as a fraction of nitrogen applied to the soil. Over time, the shift from using IPCC default emission factors to approaches incorporating Tier II and/or Tier III adjustments (Brazil, 2020) has improved accuracy. For Tier II, the weighted emission factors for mineral and mineral + organic fertilizers are 0.5% and 1.6% of nitrogen inputs in dry and humid climates, respectively. For organic fertilizers, the factors are 0.5% for dry climates and 0.6% for humid climates. For nitrogen inputs from animal urine and feces, the disaggregated default values are 0.2% in dry climates and 0.6% in humid climates. A key limitation of this approach is that the same emission factor (EF) is applied regardless of fertilizer type, soil characteristics, or land use (agriculture or pasture) across Brazil’s diverse climates. This occurs because sufficiently disaggregated activity data by soil, climate, land use, and management practices are still lacking, and available data correspond to different Tiers and resolutions (Mazzetto et al., 2020).

Although centered on Brazil, this study addresses a global challenge: improving default N₂O emission factors to enhance national greenhouse gas inventories. Agricultural powerhouses like the U.S. (Venterea et al., 2016), EU (Van der Zee et al., 2021), and China (Xu et al., 2020) are advancing region- and crop-specific factors to better reflect local conditions. This shared effort underscores the limitations of Tier I methods and positions this meta-analysis as a contribution not only to Brazil’s inventory, but to the broader international push for more precise Tier II/III estimates in agriculture.

Given the current scenario, Brazilian agribusiness is expected to continue growing, and with it, fertilizer use. Thus, this review and meta-analysis study contributes to the calculation of direct N_2_O emissions at a more detailed level, differentiating emission factors based on the types of organic and inorganic fertilizers and their combinations in production systems related to sugarcane, grains, and pastures. These specific assessments focus on (i) identifying qualitative and quantitative controlling factors (climate, biome, edaphoclimatic characteristics, soil physicochemical properties, cultivation practices, fertilizer doses and types, and excreta inputs) of EF, and (ii) proposing consistent and reliable EF for mineral and organic fertilizers used in sugarcane, grain (soybean-corn), and pasture crops, for both fertilization emissions and those generated by livestock excreta (feces and urine), and (iii) indicating national EF of N₂O for the crops and pastures evaluated.

## Materials and methods

### Systematic review

A systematic review was conducted in the Web of Science, Scopus, and SciELO databases using the following search string: “Brazil” AND “nitrous oxide emission” AND “emission factor” AND “emission factors” AND (soybean OR corn OR sugarcane OR pasture OR fertilizer).

The systematic review was conducted in two stages. The initial search (2023), including studies published up to 2024, retrieved 229 articles, of which 81 were pre-selected based on the defined protocols and reference criteria. Due to the limited number of eligible studies and the rapid growth of recent publications on the topic, a second search was conducted in 2025, following the same inclusion and exclusion criteria to ensure consistency and comparability.

**Fig. 1:**
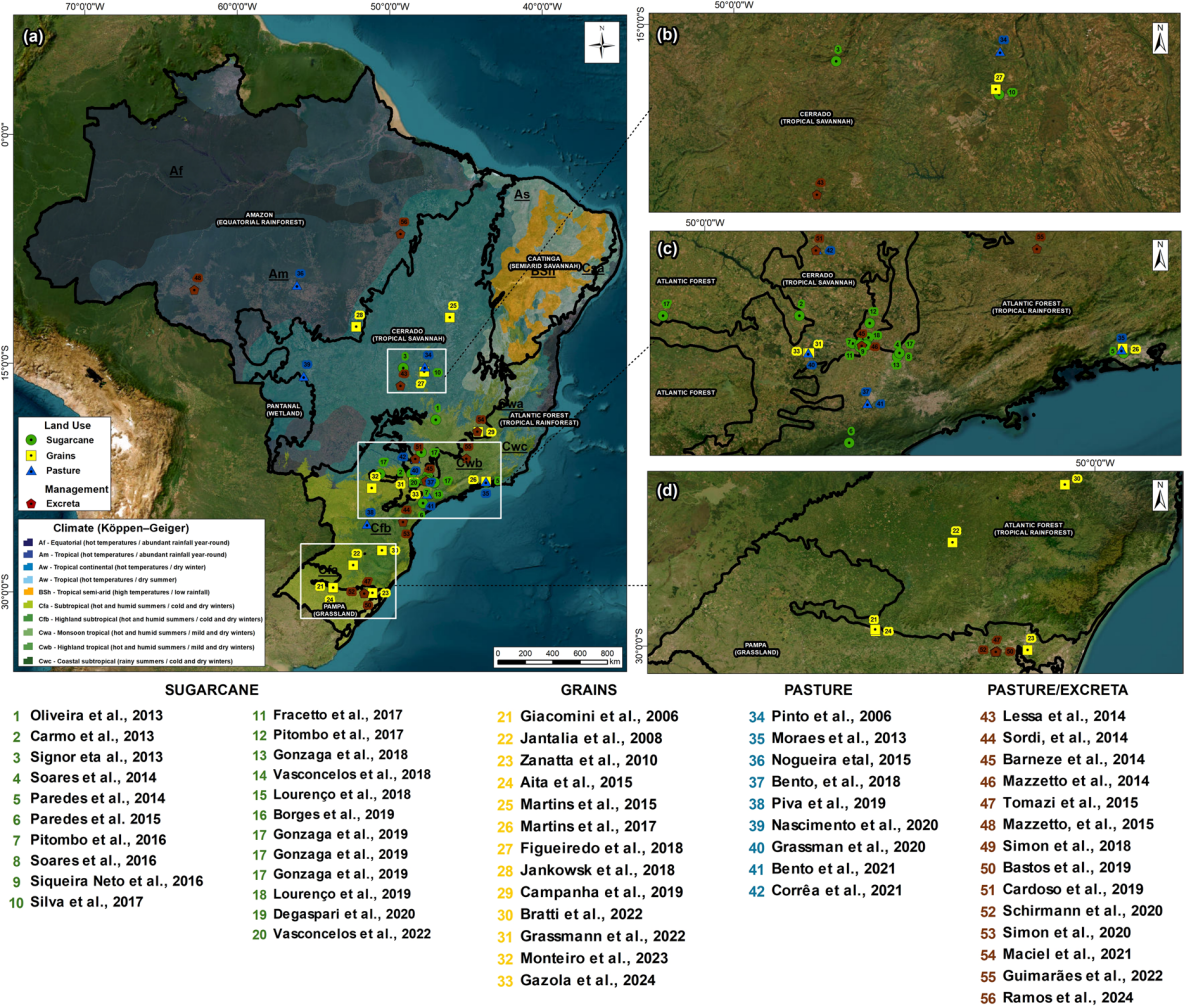
**(a)** Spatial distribution of compiled experiments on nitrous oxide (N₂O) emission factors for Sugarcane, grains, pasture and excreta, systems in Brazil, highlighting biome boundaries (IBGE, 2025) and climate classes according to the Köppen–Geiger classification (Alvares et al., 2013); Panels (**b**), (**c**), and (**d**) present zoomed-in views of regions with a higher densities of reviewed studies.

This second search identified 49 additional studies, resulting in a total of 130 articles. After a second screening round, 74 studies were excluded (e.g., laboratory-scale experiments or absence of control treatments), and 56 were considered suitable, as they provided emission factors (EFs) or sufficient data for EF estimation (Fig. 1; Table, Systematic Review -Supplementary material). The time interval between the two searches was approximately 2 years, which contributed to increasing the robustness and representativeness of the dataset.

Among the studies analyzed, 173 trials reported emission factors for sugarcane systems, 41 for grain-based systems, and 111 for pasture areas, with and without excreta deposition, totaling 325 treatments. The spatial distribution of these experiments across Brazil is shown in Fig. 1.

When accumulated N₂O emissions or emission factors (EFs) were not explicitly reported, data were extracted from tables or digitized from figures using WebPlotDigitizer (https://automeris.io/WebPlotDigitizer/index.html). For graphical data, accumulated emissions over the experimental period were estimated using the compound trapezoidal rule. All values were standardized to kg N–N₂O ha⁻^1^, and average fluxes were calculated for each treatment.

The information extracted from the articles and organized into tables was categorized into two groups: (i) qualitative variables, including soil texture, irrigation, cultivation practices, mineral N source, organic N source, and combined organic + mineral N source obtained from the literature review; and (ii) quantitative variables (13 continuous variables), including experiment duration (days), precipitation during the experimental period (mm), mean temperature (°C), clay (%), silt (%), sand (%), pH (CaCl₂), organic matter (OM; g dm⁻^3^ or g kg⁻^1^), soil carbon and nitrogen (g kg⁻^1^), mineral N dose (kg ha⁻^1^), organic N dose (kg ha⁻^1^), and combined mineral + organic N dose (kg ha⁻^1^).

This dataset was further complemented with covariates (moderators), including climate classification according to Köppen–Geiger (Alvares et al., 2013), biome (IBGE, 2025), and soil classification based on the Brazilian Soil Classification System (Embrapa, 2018) and in the World Reference Base (WRB, 2022), obtained through GIS analysis. All geoprocessing analyses were performed using ArcGIS, and statistical analyses were conducted in R version 4.3.2 (R Core Team, 2023).

Of the 56 studies, 42 reported calculated N₂O EFs, and 10 had their EF values calculated based on Eq. (1) and the data provided in the studies.

1 $$FE_{N2O}=\left(\frac{N_{N2O\ treatment}-N_{N2O\ control}}{N_{applied\ (fertilizer)}}\right)\times100$$

Where $FE_{N2O}$ is the emission factor (N–N₂O expressed as a percentage of the N applied as fertilizer); N–N₂O treatment and N–N₂O control represent the N–N₂O emissions from fertilized and unfertilized treatments, respectively; and N applied (fertilizer) is the amount of N applied to the system. The N₂O emission factors (EFs) obtained for the main management systems of sugarcane, grain crops, and pastures were considered dependent variables.

For sugarcane, the trials were classified into three categories based on cultivation practices: unburned cane with low mineral + organic fertilization (below 90 kg of N ha^−1^), unburned cane with moderate fertilization (between 90 and 180 kg of N ha^−1^) and unburned cane with high fertilization (above 180 kg of N ha^−1^).

Emission factors from trials involving mineral or organic fertilizers combined with nitrification inhibitors (NI) or controlled-release formulations (CRF) were excluded from the statistical analyses. These additives and technologies fundamentally alter the kinetics of nitrogen transformation in the soil, such as by delaying nitrification or modulating nitrogen release, resulting in emission factors that are not directly comparable to those derived from conventional fertilizers, which are the primary focus of this national-scale assessment. Likewise, studies incorporating sugarcane burning were excluded to isolate the effects of fertilization from the confounding influence of fire on nutrient cycling and greenhouse gas emissions.

Emission factors from different seasons within the same trial were retained to account for climate-driven interannual variability. To avoid pseudoreplication, the meta-analysis employed bootstrapping at the level of entire experimental units (defined by study, location, and treatment), rather than individual seasonal observations, ensuring that the resulting confidence intervals accurately reflect the hierarchical structure of the dataset.

### Statistical treatment

Descriptive analyses were initially performed using boxplots to explore data distribution and identify patterns among variables. For each median, 95% confidence intervals were calculated to support comparisons among factor levels based on interval overlap.

Differences in emission factors across categorical variables were assessed using the nonparametric Kruskal–Wallis test, which compares group medians based on ranked data; the results are presented in Table S1. This approach was selected due to the non-normal distribution and heteroscedasticity observed in the dataset, making it more robust than parametric alternatives.

Linear regression models were applied to evaluate relationships between emission factors (response variable) and continuous predictors (moderators), including climatic, soil, and management variables. Model assumptions, linearity, independence of residuals, homoscedasticity, and normality of residuals, were assessed through diagnostic plots. When necessary, data transformations were considered to improve model fit.

### Meta-analysis

A meta-analysis was performed to assess the effects of environmental moderators on the N₂O emission factor, defined as a “measure of association” representing the difference in means between a treatment and its control. In general, this measure is weighted by the reciprocal of the variance, assigning greater weight to experiments with higher precision. However, only half of the selected articles reported measures of variation. Consequently, a method without variance weighting was applied (Dong et al., 2018). In this approach, means and 95% confidence intervals for the emission factor were estimated using a nonparametric bootstrap-resampling method with 10,000 iterations, implemented through the BootES package (Gerlanc & Kirby, 2021).

The weighted emission factor (WEF) was calculated to account for variation in sample size across experiments, using a formulation analogous to a weighted harmonic mean. Each emission factor (EF_i) was normalized by its number of observations (n_i), and the overall weighted value was derived using Eq. 2:

2 $${\boldsymbol{W}}{\boldsymbol{E}}{\boldsymbol{F}} = (\sum \_(i=1)^N (EF\_i/n\_i)) / (\sum \_(i=1)^N (1/n\_i))$$

where ***WEF*** is the weighted emission factor; ***EFₙ*** is the emission factor from study ***i***; ***nₙ*** is the number of observations (or replicates) in study ***i***; and ***N*** is the total number of studies.

This approach uses inverse sample size weighting (1/nᵢ), preventing studies with large datasets from dominating the estimate. It ensures a more balanced contribution among experiments, improving robustness across heterogeneous datasets.

Differences between estimates and a specific value, or between levels of qualitative moderators, were assessed by examining the overlap of 95% confidence intervals. The significance of the linear relationship between the emission factor and the quantitative moderators was also evaluated, corresponding to the slope estimate in regression analysis. A significance level of 5% was adopted.

## Results

IPCC emission factors are essential coefficients used to estimate greenhouse gas emissions from human activities. They support national inventories, enable global comparisons, and inform climate policy development. The IPCC’s Emission Factor Database (EFDB) improves accuracy by consolidating international data. However, the default emission factors do not adequately reflect the environmental conditions of many countries. Therefore, developing region-specific factors, particularly for diverse soils and climates, is crucial for enhancing agricultural impact assessments and designing sustainable, locally adapted practices, to complement the EFDB.

### N_2_O emission factors in tropical and subtropical agricultural systems in Brazil

**Fig. 2:**
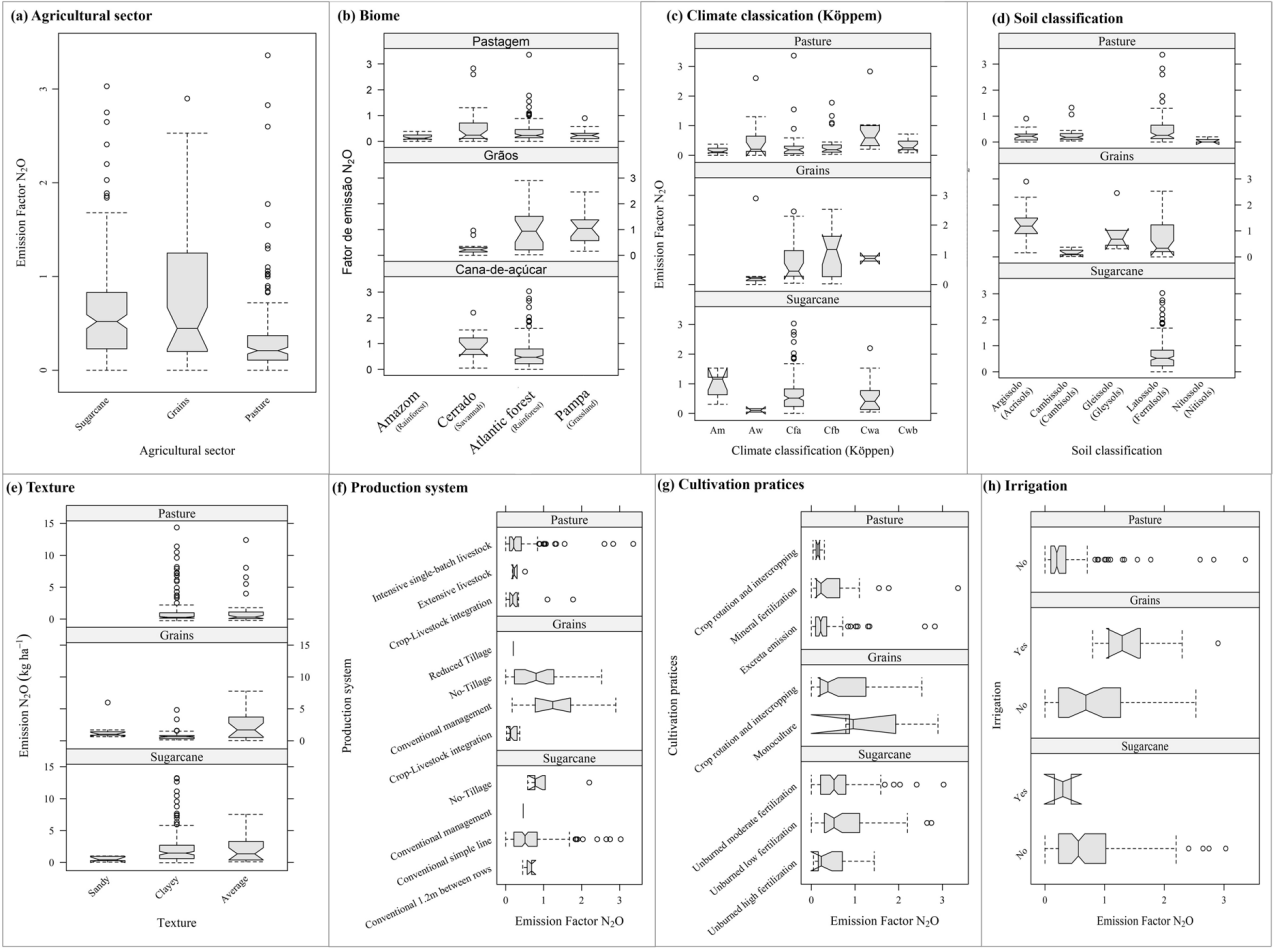
Distribution of values (*box plot*) and EF of N_2_O in pasture, grain and sugarcane systems, under the effects of (**a**) land use, (**b**) biome, (**c**) climate, (**d**) soil classification, (**e**) soil texture, (**f**) production system, (**g**) cultivation practice, and (**h**) irrigation practice. Köppen climate classes: Am (tropical monsoon), Aw (tropical savanna), Cfa (humid subtropical), Cfb (temperate oceanic), Cwa (subtropical with dry winter), Cwb (subtropical highland)

**Fig. 3:**
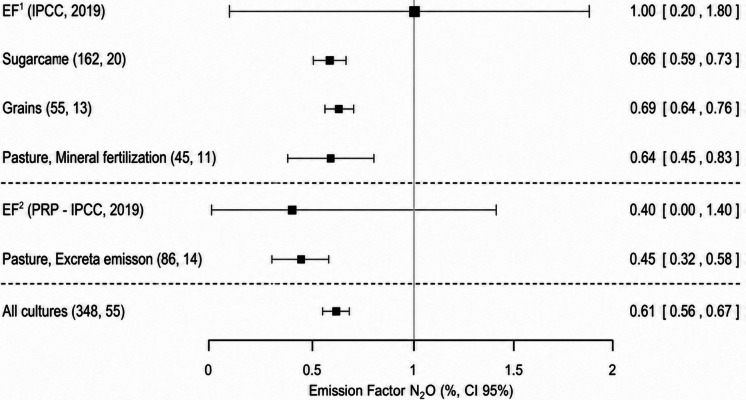
IPCC default values and weighted average, minimum, and maximum N₂O emission factors (EF) by land use in sugarcane, grain, and pasture production systems. Values in parentheses indicate the number of treatments and the number of studies, respectively, while values in brackets represent the range of emission factors across land uses. EF^2^ denote IPCC (2019) default emission factors for fertilization and excreta deposition, respectively; PRP, pasture, range, and paddock; IPCC, Intergovernmental Panel on Climate Change

The distribution of values (boxplots) and N₂O emission factors (EFs), under the influence of climate, biome, soil classification and texture, and cultivation practices, is presented in Figs. 2 and 3, Table 1, and Supplementary Table S1, where the Kruskal–Wallis test results are reported. In this analysis (Fig. 2), averages were tested without weighting. Higher mean EF values for pasture systems were observed in the Cerrado and Atlantic Forest biomes, while grain systems exhibited higher EF values in the Atlantic Forest and Pampa biomes, and sugarcane systems in the Cerrado and Atlantic Forest biomes (Fig. 2b). Regarding climate conditions, pasture systems generally showed higher EF values under Cwa climatic conditions, followed by Aw and Cwb climates. Grain systems presented high and highly variable EF values under both subtropical (Cfb and Cfa) and tropical climates, indicating overlapping emission patterns among climatic classes (Fig. 2c). Sugarcane systems showed the highest EF values under Am and Cfa climates, although intermediate climatic classes exhibited overlapping distributions.

**Table 1:** **N**_2_O emission factors for different fertilizer types across sugarcane, grain, and pasture production systems in Brazil

Use	Fertilizer	Emission (kg ha ^-1^)	(EF) (%) (IC)	n° of treatments	n° of references	References used^1^
Sugarcane	Ammonium nitrate	1.9 (1.69–2.14)	0.47 (0.41–0.54)	44	14	2, 3, 7, 8, 9, 10, 11, 12, 15, 16, 17, 18, 19, 37
Sugarcane	Ammonium nitrate + organics	4.52 (3.63–5.45)	0.94 (0.71–1.18)	35	5	2, 7, 15, 17, 18
Sugarcane	Straw	2.04 (1.79–2.46)	0.38 (0.22–0.53)	6	2	12, 14
Sugarcane	Ammonium sulfate	4.1 (3.84–4.42)	0.44 (0.36–0.54)	11	3	6, 13, 17
Sugarcane	Ammonium sulfate + organics	3.9 (3.86–3.95)	0.63 (0.56–0.73)	12	3	6, 17, 20
Sugarcane	Filter cake	1.13 (1.12–1.15)	0.14 (0.1–0.17)	2	1	9
Sugarcane	Urea	2.34 (1.93–2.75)	0.95 (0.88–1.04)	21	7	2, 3, 4, 5, 8, 9, 19
Sugarcane	Urea + organics	2.05 (1.65–2.4)	1.08 (0.73–1.52)	5	2	2, 9
Sugarcane	Vinhasse	1.24 (1.14–1.37)	0.93 (0.76–1.1)	30	7	1, 5, 6, 7,9, 15, 18,
Grains	Pig manure	4.52 (2.98–6.16)	1.35 (1.01–1.78)	6	1	24
Grains	Minerals + crop residues	0.73 (0.65–0.84)	1.63 (1.29–2.03)	8	1	22
Grains	Minerals	1.93 (1.71–2.15)	0.66 (0.6–0.73)	35	11	23, 24, 25, 26, 27, 28, 29, 30, 31, 33, 32
Grains	Crop residues	0.7 (0.65–0.75)	1.34 (0.86–1.83)	4	1	22
Grains	Crop residues + pig manure	0.45 (0.4–0.51)	0.18 (0.16–0.2)	2	1	21
Pasture	Minerals	0.70 (0.53–0.87)	0.64 (0.45–0.84)	45	11	30, 34, 35, 36, 37, 38, 39, 40, 41, 42, 51
Pasture	Feces + urine	0.68 (0.45–0.91)	0.40 (0.26–0.54)	4	2	51, 56
Pasture	Feces	1.13 (1–1.27)	0.15 (0.13–0.16)	35	10	43, 44, 46, 47, 49, 50, 51, 52, 54, 55
Pasture	Urine	4.62 (4.06–5.22)	0.73 (0.62–0.84)	47	12	43, 44, 45, 47, 48, 49, 50, 51, 52, 53, 54, 55

^1^The numbers listed in the “References Used” column correspond to the respective sources cited in Fig. 1. EF - Emission Factors; CI - Confidence interval

Latosols (Ferralsols) showed higher mean emission factors for pastures, possibly associated with their well-drained structure and high permeability, which may favor nitrification processes. However, their low natural fertility and high acidity can also limit nitrogen availability. In contrast, Argisols (Acrisols/Ultisols) exhibited higher emission factors in grain production systems (Fig. 2d). Nevertheless, the dataset was strongly concentrated in these soil classes, limiting broader comparisons with other soil types.

Soil texture showed only a limited influence on emission factors (Fig. 2e), although medium-textured soils tended to present higher EF values in grain and sugarcane systems. The results for cultivation systems and management practices (Fig. 2f and g) were generally inconclusive due to high variability and overlapping statistical groups. Weighted emission factors calculated for pasture and grain systems using mineral and organic fertilizer input data (Table 1) showed substantially different variances and statistically significant differences among agricultural systems. In sugarcane systems, higher weighted EFs were associated with urea + organics, urea, ammonium nitrate + organics, and vinasse applications, whereas lower values were observed for filter cake and straw management. In grain systems, the highest weighted EFs were associated with mineral fertilizers combined with crop residues and pig manure application. For pastures, urine depositions showed higher weighted EF values than feces or mixed excreta depositions.

Irrigation tended to increase N₂O emission factors in grain systems, while weaker or more variable responses were observed in pastures and sugarcane systems (Fig. 2h).

Table 1 and Fig. 3 present the weighted average N₂O emissions and emission factors (EFs) for pasture, grain, and sugarcane systems. These results summarize EFs according to different fertilizer and management categories, including mineral and organic fertilizers and, in pasture systems, excreta deposition. The weighted averages were calculated according to the number of trials per study. The distribution of observations and statistical comparisons among environmental and management categories are presented in Fig. 2 and Supplementary Table S1, respectively.

### N_2_O emission factors: influence of environmental conditions and soil management

**Fig. 4:**
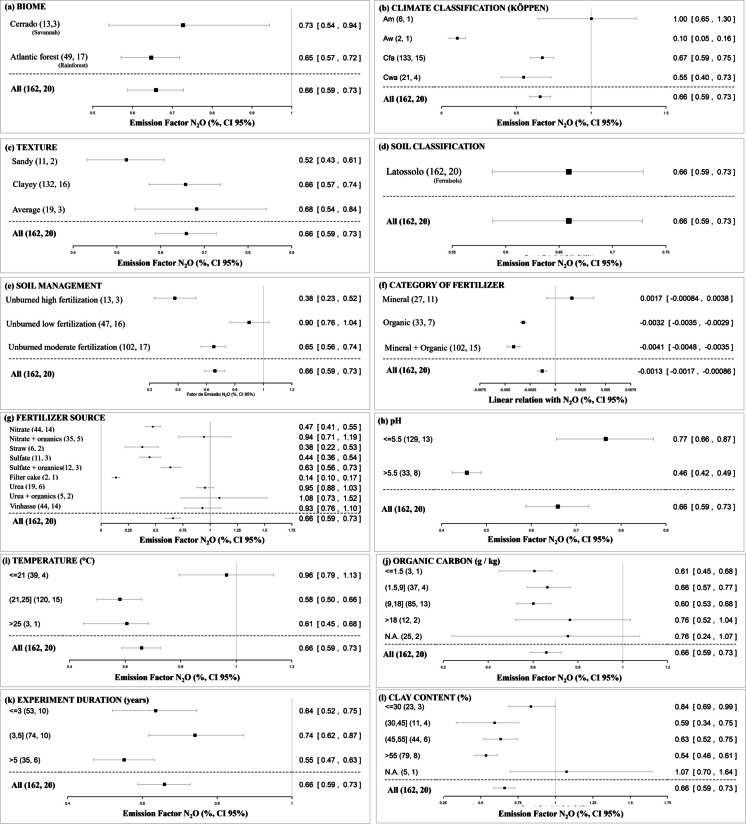
Weighted average values of EF of N_2_O in sugarcane cultivation systems, under the effects of (**a**) biome, (**b**) climate, (**c**) granulometry, (**d**) soil class, (**e**) management, (**f**) fertilizer type, (**g**) fertilizer source, (**h**) pH, (**i**) temperature, (**j**) organic carbon, (**k**) experimental time, and (**l**) clay content. The data in parentheses indicate, respectively, the number of treatments evaluated, and the number of articles reviewed, while values in brackets represent the range of confidence interval (95%) of emission factors identified across different land uses in the review

**Fig. 5:**
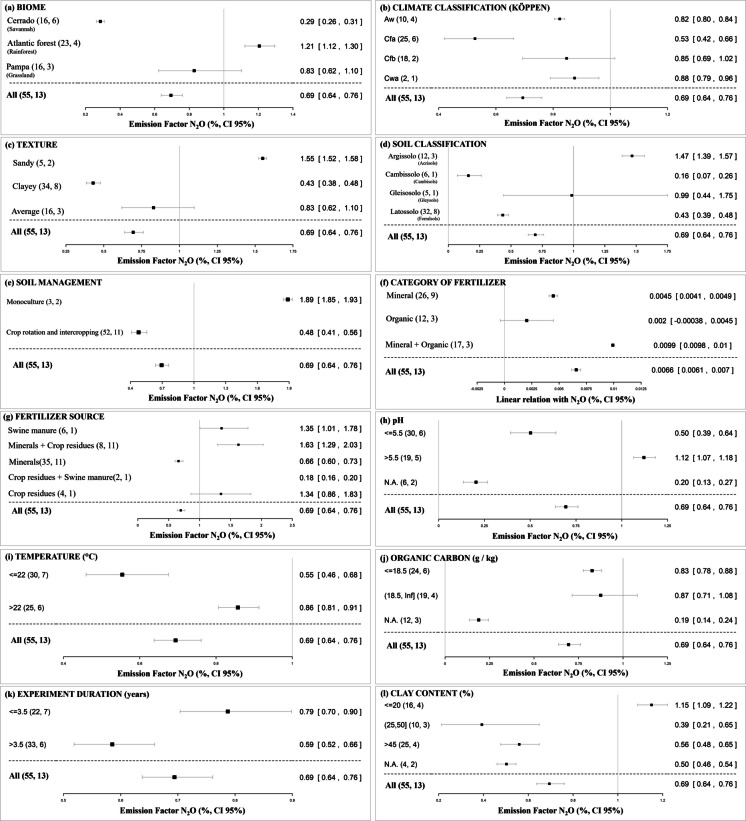
Average weighted values of EF of N_2_O in grain cultivation systems, under the effects of (**a**) biome, (**b**) climate, (**c**) granulometry, (**d**) soil class, **e** management, (**f**) fertilizer type, (**g**) fertilizer source, (**h**) pH, (**i**) temperature, (**j**) organic carbon, (**k**) experimental time, and (**l**) clay content

**Fig. 6:**
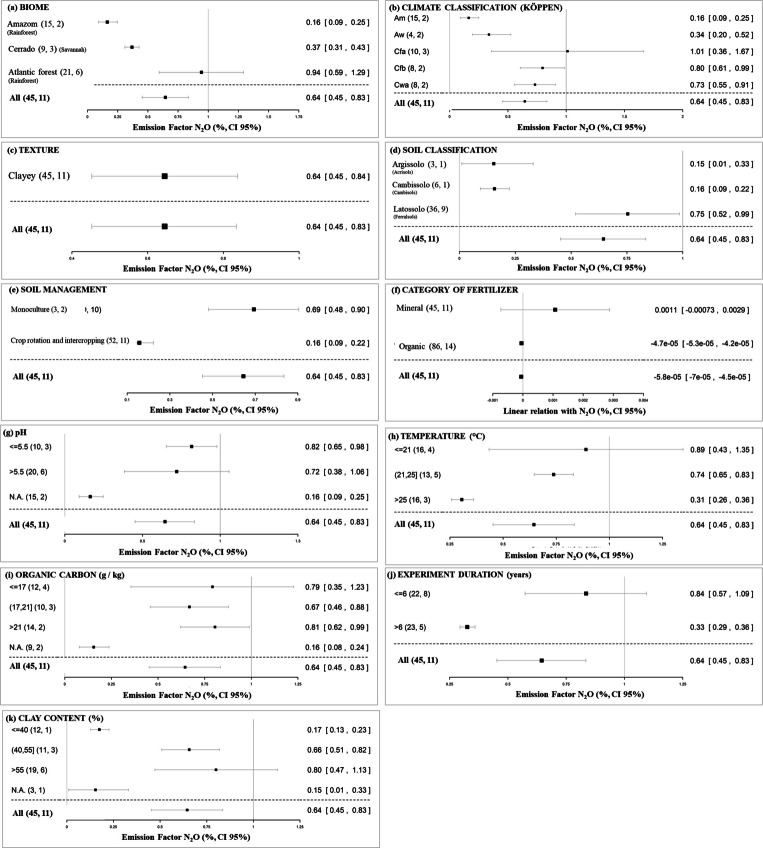
Average weighted values of EF of N_2_O in livestock systems on mineral-fertilized pastures without the presence of cattle excreta, under the effects of (**a**) biome, (**b**) climate, (**c**) granulometry, (**d**) soil class, (**e**)fertilizer source (here the type of fertilizer is only mineral), (**h**) pH, (**i**) temperature, (**j**) organic carbon, (**k**) experimental time, and (**l**) clay content

**Fig. 7:**
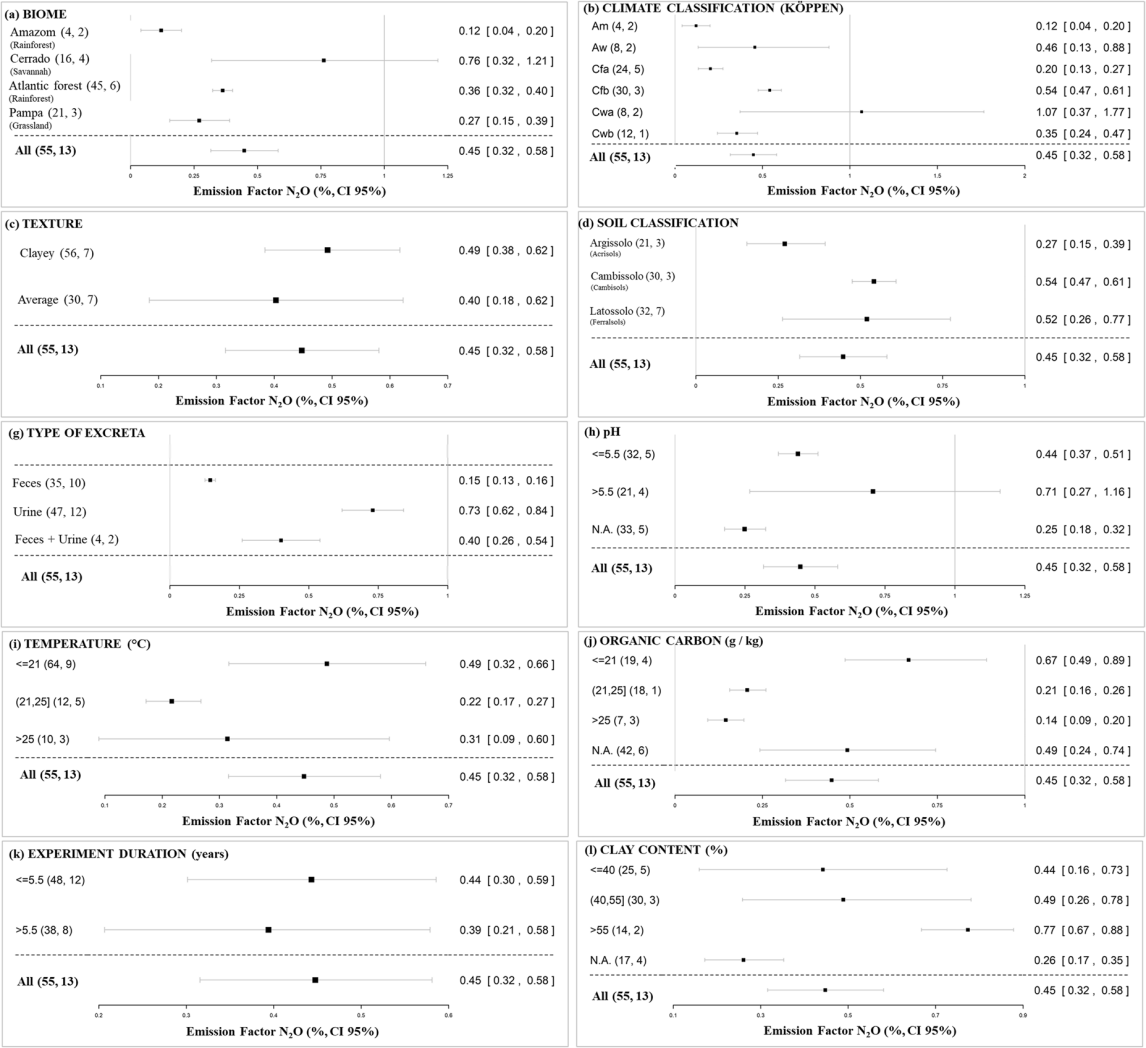
Average weighted values of EF of N_2_O in livestock systems in pastures with the presence of cattle excreta, under the effects of (**a**) biome, (**b**) climate, (**c**) granulometry, (**d**) soil class, (**e**) type of excreta, (**f**) pH, (**g**) temperature, **h** organic carbon, (**i**) experimental time, and (**j**) clay content

The aggregated emission factors by crop reflect the combined influence of multiple controlling factors, particularly nitrogen inputs from mineral and organic sources. Emission levels varied according to climatic and soil conditions, as well as land use and management practices. Figures 4, 5, 6, and 7, together with Supplementary Table S1, present the emission distributions obtained using different environmental data grouping approaches.

Overall, experiment duration influenced emission factors, with shorter experiments generally showing higher EF values. This pattern likely reflects the concentration of measurements during peak emission periods following fertilization and rainfall events, which may overestimate cumulative emissions, whereas longer experiments better capture temporal variability and tend to provide more representative average values.

The highest N₂O emission factors for sugarcane (Figs. 2a and 3; Supplementary Table S1) were observed in the Cerrado and Atlantic Forest biomes (Fig. 4a), under Am and Cfa climates (Fig. 4b), and in medium-textured soils (Fig. 4c, d). These conditions were generally associated with unburned sugarcane systems receiving low to moderate fertilizer applications (Fig. 4e). Mineral fertilizers were generally associated with higher EF values than organic sources, particularly when combined with organic amendments (e.g., ammonium nitrate + organics and urea + organics), which tended to further increase emissions (Fig. 4f). More acidic soil conditions were also associated with higher EF values, as were increasing soil organic carbon contents (Fig. 5h). In addition, lower temperatures were associated with higher EF values, suggesting complex interactions among climatic and soil factors regulating N₂O emissions (Fig. 5i). It is important to highlight that the Atlantic Forest contains the largest share of sugarcane cultivation area in Brazil, followed by the Cerrado, contributing to the greater density of observations in these biomes (Fig. 1).

Linear relationships between fertilizer type (mineral, organic, and mineral + organic) and EF showed distinct response patterns. A 100-kg increase in fertilizer application was associated with an increase of approximately 0.2% in EF for mineral fertilizers, whereas decreases of approximately 0.4% and 0.5% were observed for organic and combined applications, respectively (Fig. 4f; Supplementary Fig. 1). In contrast, among the qualitative variables, soil nitrogen and silt contents showed positive relationships with EF, indicating a positive association with N₂O emissions (Supplementary Fig. 6).

The highest grain-crop emission factors (Figs. 2a and 3; Supplementary Table S1) were observed in the Atlantic Forest and Pampa biomes (Fig. 5a), under Cfb climate conditions (Fig. 5b), and in medium-textured Argisols (Fig. 5c, d), particularly under monoculture systems (Fig. 5e). Emissions tended to be higher under monoculture and conventional management systems, especially where crop residues and pig manure were applied, as well as under higher temperatures and soil pH values above 5.5 (Figs. 5g–i). Medium-textured soils were associated with higher EF values than clayey and sandy soils (Fig. 5l). It is important to note that the Cerrado contains the largest share of grain production in Brazil, followed by the Atlantic Forest, which contributes to the greater concentration of observations in these regions (Fig. 1).

The linear relationships between mineral, organic, and combined mineral-organic fertilizers and EF showed that applying a 100-kg dose of each in grain systems increased emission factors by 0.3%, 0.2%, and 1%, respectively (Fig. 5f and Supplementary Fig. 2). Among qualitative variables, pH and nitrogen content were significantly and positively associated with EF (Fig. 5h and Supplementary Fig. 2).

In pastures with mineral fertilization (Figs. 2a and 6, Supplementary Table S1), the highest emission factors were observed in the Atlantic Forest and Cerrado biomes (Fig. 6a), under Cwa climatic conditions (Fig. 6b), and on clayey Ferralsols (Latosols) (Figs. 6c,d). Emissions increased under continuous grazing (i.e., without pasture rotation) (Fig. 6e), more acidic soils (Fig. 6h), and higher organic carbon content (Fig. 6g), and were also associated with lower temperatures (Fig. 6i). Silt, clay, and organic matter exhibited significant positive relationships with EF (Fig. 6l and Supplementary Fig. 3).

The highest emission factors in pastures with excreta (Figs. 2a, 3, and 7, Supplementary Table S1) were observed in the Cerrado and Atlantic Forest biomes (Fig. 7a), under a subtropical climate with a dry winter (Cwa) (Fig. 7b), and on clayey Ferralsols (Latosols) and Cambisols (Fig. 7c, d). Higher emissions were also associated with lower soil pH (Fig. 7h) and lower temperatures (Fig. 7i). Urine presented higher emissions than feces (Fig. 7g). Only pH was significantly and positively associated with EF (Supplementary Fig. 4).

The linear relationship between mineral fertilizer and EF indicated that applying a 100-kg dose of mineral fertilizer increased the pasture emission factor by 0.1% (Fig. 4, supplementary material). The weighted average N₂O EF values in pasture systems were 0.64 for mineral fertilization, 0.15 for feces, 0.40 for urine, and 0.73 for feces + urine, resulting in an average EF of 0.51 for excreta (Table 1).

It is important to highlight that pasture systems are extensively distributed across Brazil, with a higher concentration in the Cerrado and Amazon biomes. In contrast to other agricultural land uses, pasture is present in all regions and biomes, resulting in a more widespread spatial distribution of observations (Fig. 1).

Finally, linear regression models were developed (Figs. 5 and 6, supplementary material). Although these models showed low explanatory power, the results were included to emphasize the need for further research on N₂O emissions, given the wide range of management practices and soil and climate conditions that affect this natural process. In the generated models, soil factors such as organic matter (OM) and carbon (C) (Fig. 5g and f, supplementary material) influenced emission factors in pastures.

Linear regression analysis revealed that the mineral + organic dose (Fig. 6, supplementary material) significantly influenced grain emission factors (*p* < 0.05). For grains, soil sand content, organic matter (OM), and nitrogen (Fig. 5d, f, and h) also showed significant effects (*p* < 0.05). In sugarcane systems, temperature, clay, sand, and OM (Fig. 3a, d, and f) were strongly associated with emission factors (*p* < 0.01).

## Discussion

The distribution of N₂O emission factors (EFs), influenced by climate, biome, soil (class and texture), and management practices, is presented in Figs. 2 and 3 and Table 1. In this analysis (Fig. 2), averages were tested without weighting. Higher EF values were observed in pastures within the Cerrado and Atlantic Forest, while for grains the highest values occurred in the Atlantic Forest and Pampa, and for sugarcane in the Cerrado and Atlantic Forest (Fig. 2b). These patterns are likely related to higher precipitation in the Atlantic Forest, influenced by the Atlantic Ocean, and to intense rainfall events in the Cerrado, often associated with fire use (Rios et al., 2024). Prolonged drought periods reduce vegetation vigor, increase fire spread (MapBiomas, 2023), and enhance soil vulnerability to erosion (Trindade et al., 2016), amplifying environmental impacts and reinforcing the need for sustainable management.

In general, emissions increased under more acidic soil conditions, higher organic carbon content, and lower temperatures. Acidic conditions can influence microbial pathways, often favoring incomplete denitrification and greater N₂O accumulation. Higher organic carbon provides substrate for microbial activity, while moderate temperatures may limit the reduction of N₂O to N₂, resulting in higher emissions.

Most sugarcane trials were conducted in tropical and subtropical climates, mainly in the Atlantic Forest and on Latosols with medium to clayey textures. A few trials in the Caatinga showed high EF values, mainly linked to urea topdressing (Lopes et al., 2018); however, these values were excluded due to incompatibility with the reviewed dataset, likely reflecting specific experimental conditions and limited regional representativeness. The highest EFs were observed in tropical climates (Am and Cfa; Fig. 2), while grain crops in subtropical climates (Cfb and Cfa) showed values comparable to tropical systems (Fig. 2c; Table 1). This reflects the strong influence of maritime air masses in Brazil, which promote more uniform rainfall distribution, contrasting with continental temperate climates where precipitation is more seasonal (Barry & Chorley, 2013). This difference highlights the limitations of applying IPCC Tier I factors (IPCC, 2006), developed under Northern Hemisphere conditions.

The uncertainty in EF estimates, reflected in wide confidence intervals, arises from the inherent variability of N₂O fluxes, the limited number of trials for specific categories (e.g., filter cake), and the data extraction process. While bootstrapping accounts for sample variability, this uncertainty is intrinsic to national EF estimation. Thus, the EFs represent robust averages within a probable range, supporting their application in policy frameworks such as RenovaBio (Brazil, 2017). Emission factors stratified by land use (Fig. 3), management (Table 1), and environmental conditions (Fig. 2), and adjusted to national variability (Figs. 4, 5, 6, and 7), improve the assessment of fertilization systems and mitigation strategies. When compared with IPCC references (Fig. 3), these results provide a more accurate basis for emissions accounting and environmental compensation mechanisms.

N₂O formation in soils is primarily driven by nitrification and denitrification processes, influenced by moisture, temperature, oxygen availability, and C and N dynamics. These factors can be modified by management practices such as no-till, residue management, fertilization, and animal excreta. Differences in agronomic practices among pastures, grains, and sugarcane explain variations in emissions (Ayer et al., 2015; Bertol et al., 2019; Duku et al., 2025; Ghosh et al., 2024; Pophiwa et al., 2025; Pulido-Moncada et al., 2022; Ritchie & Roser, 2021; Vasconcelos et al., 2018).

These integrated results provide the basis for the following analysis of N₂O emissions in the main agricultural and livestock systems in Brazil, focusing on the impacts of fertilizers and management practices in sugarcane and grain cultivation and pasture systems, as described in the sections.

###  N_2_O emissions in sugarcane cultivation: impacts of fertilizers and agricultural techniques

According to a review by Mazzetto et al. (2020), which analyzed seven studies on sugarcane, two on pastures, and two on corn, the IPCC’s current default emission factor for N₂O (1%) is lower than the average value reported in their compilation (1.12%). The review also indicated that fertilizers without urea, such as ammonium nitrate and ammonium sulfate, resulted in lower N₂O emission factors, at 1.07% and 0.60%, respectively.

Consistent with previous findings, the values compiled in this study indicate an emission factor (EF) of 0.95 for urea in sugarcane trials (Table 1). This contrasts with Lopes et al. (2018), who reported higher EFs when nitrogen was applied via irrigation water, a method rarely adopted in commercial sugarcane production. Among the 16 trials analyzed, most involved unburned sugarcane under topdressing with straw retained on the soil, a condition known to enhance N₂O emissions. In contrast, EFs for ammonium nitrate and ammonium sulfate were considerably lower, at 0.46 and 0.44, respectively. Notably, approximately 70% of the trials using these fertilizers also maintained straw cover, suggesting that fertilizer type plays a more decisive role than residue management alone.

In a review of studies on N₂O emission factors (EF) in sugarcane areas (16 evaluations, 60 trials), Vasconcelos et al. (2022) reported an average EF of 0.63 for combined nitrogen sources and 1.37 for exclusive vinasse applications. In the present review, the average EF for combined mineral fertilizers was 0.72, similar to that reported by Vasconcelos et al. (2022). However, the EF for vinasse was lower, at 0.87. While Vasconcelos et al. (2022) analyzed 9 trials involving exclusive vinasse application, this study included those same trials plus an additional 19, which resulted in a different average (Table 1, supplementary material).

Sugarcane plays a central role in the Brazilian economy (Cardoso et al., 2019; Mello et al., 2014; Oliveira et al., 2018), with sugarcane ethanol being the most prominent biofuel in the country’s energy matrix (Karp et al., 2021). The rapid expansion of sugarcane cultivation (Mello et al., 2014; Brazil, 2020) has established sugarcane as Brazil’s primary renewable energy source, positioning the country as a global leader in ethanol production as an alternative energy option (Heinrichs et al., 2017).

Brazil currently accounts for 40% of global sugarcane production (Barbosa et al., 2021; FAO, 2021; Meghana & Shastri, 2020), with an average productivity of approximately 75.6 Mg ha⁻^1^ in 2020 (CONAB, 2021; FAO, 2021). In the same year, the country produced 24.8 billion liters of bioethanol (CONAB, 2021) from 8.3 million hectares of harvested sugarcane (CONAB, 2022a, 2022b). Global demand for sugarcane and its valuable co-products is projected to continue increasing (Figueroa-Rodríguez et al., 2019), and sugarcane cultivation in Brazil is expected to expand to 18.8 million hectares by 2050 (Soltangheisi et al., 2019, 2021).

According to Picoli et al. (2009), sugar and ethanol production largely depends on the amount of raw material available for milling, which is influenced, among other factors, by the size of the cultivated area, a dynamic that can drive increased land conversion to sugarcane fields. Adami et al. (2012) sought to quantify the direct land-use changes that occurred during the first decade of the twenty-first century, driven by rising demand for sugarcane for ethanol production, and found that sugarcane expanded as follows: 69.7% over pastures, 25.0% over annual crops, 0.6% over forests, and 3.4% from areas under sugarcane crop rotation. The Fourth National Inventory of Greenhouse Gas Emissions (Brazil, 2020) also reported a reduction in forest conversion to anthropogenic uses (such as pasture and agriculture) between 2010 and 2016, while highlighting a significant increase in the conversion of pastures to croplands during this period. Considering the projected expansion of cultivated areas in the country (Soltangheisi et al., 2019, 2021), it is crucial to assess the impacts of land-use change and management practices on the carbon balance.

In Brazil, the practice of burning sugarcane straw before harvest, previously used to facilitate manual cutting, has been largely replaced by mechanized harvesting over the past decade, particularly in the Center-South region, which accounts for more than 95% of the country’s planted area. This shift has resulted in the accumulation of large amounts of straw on the soil surface, requiring innovative management strategies (Bordonal et al., 2018; Carvalho et al., 2017; Soltangheisi et al., 2021). Such changes can significantly influence nitrogen dynamics.

Some studies conducted in Brazil have assessed the impact of nitrogen fertilization on N₂O emissions in sugarcane crops. Some reported emission factors below the IPCC default value (Gonzaga et al., 2018; Vasconcelos et al., 2022), while others found values above it (Mazzetto et al., 2020). The nitrogen source used is another critical factor: several studies have observed a marked increase in N₂O emissions with urea application, particularly when applied as topdressing and in the presence of straw (Carmo et al., 2013; Lopes et al., 2018). The effects of straw and other organic amendments, such as vinasse, filter cake, and bagasse, have also been investigated, as they affect soil moisture, temperature, and microbial activity, and provide readily mineralizable carbon (C) and organic nitrogen (N), among other factors. These findings are consistent with the higher EFs observed in sugarcane systems located predominantly in the Atlantic Forest and Cerrado biomes, under Am and Cfa climates, and managed under unburned systems with low to moderate fertilizer inputs.

Sugarcane in São Paulo is mostly cultivated conventionally (plowing), with soil prepared every 5 years and over 95% of fields harvested without burning. Although nitrogen fertilizers like urea are applied to sugarcane straw, there are questions about urea’s effectiveness and whether alternative fertilizers should be used. Optimal nitrogen management affects both productivity and gaseous N losses (Degaspari et al., 2020).

### N_2_O emissions in grain cultivation—soybeans and corn: impacts of fertilizers and agricultural techniques

Brazil’s grain production reached 252.7 million tons in the 2020/21 harvest, with soybeans and corn contributing 137.6 and 87 million tons, respectively, together accounting for 88% of total output (CONAB, 2022b). These two crops generated approximately R$536.5 billion of the R$1.20 trillion in gross production value from Brazilian agriculture, which currently represents about 15% of the country’s GDP (CNA, 2022).

Soybeans and corn are currently the two most important crops in Brazil in terms of harvested area, occupying 38 million and 15.7 million hectares, respectively (CONAB, 2022b, 2025). Among all crops, corn has the highest consumption of synthetic nitrogen (N) fertilizers in Brazilian agriculture, accounting for approximately 25% of total use, most of it in the form of urea (ANDA, 2021). While urea is economically attractive, its use raises agronomic and environmental concerns due to nitrogen losses. These losses can contribute significantly to direct N₂O emissions through nitrification and denitrification, as well as to indirect emissions via ammonia (NH₃) volatilization. One strategy to mitigate the higher emissions associated with nitrogen fertilization has been the widespread adoption of conservation practices, such as no-till farming systems (Jantalia et al., 2008; Metay et al., 2007) and integrated crop-livestock-forestry systems (Amadori et al., 2022).

Grain and sugarcane crops are classified as annual and semi-perennial, respectively. Grains can be cultivated using either conventional or no-till systems, which differ in the intensity of soil disturbance and the amount and persistence of crop residues. The adoption of mechanical, soil, and vegetative management practices can help mitigate some of the impacts related to N₂O emissions in these cropping systems (Campanha et al., 2019).

Among these practices, the no-till system (NTS), introduced in Brazil in the 1960 s, had expanded to approximately 33 million hectares by 2017. Of this area, 77.6% was concentrated in the Central-West and South regions. The annual rate of NTS adoption was around 1.6 million hectares during the 1990 s, declining to 1.2 million ha/year between 2001 and 2008, and further dropping to 0.3 million hectares per year in the current decade. This slowdown was expected, as no-till is now widely adopted, particularly within integrated crop-livestock-forestry systems (ILPF Network, 2021).

Campanha et al. (2019) reported that the no-till system (NT) reduced N₂O emissions by approximately 30% compared to the conventional tillage system (CT) during the crop growing season. In contrast, the application of urea significantly increased emissions, by up to tenfold, highlighting its substantial environmental impact. On an annual basis, cumulative N₂O emissions under no-till were lower, reaching 4.06 kg ha⁻^1^, compared to 5.06 kg ha⁻^1^ under conventional tillage. These findings underscore the potential of no-till systems to mitigate greenhouse gas emissions in agricultural systems. However, in the present study, grain-crop EFs were highest under conventional management and no-tillage systems, whereas crop-livestock integration systems presented substantially lower EFs (Supplementary Table S1), highlighting the importance of integrated management practices.

Therefore, adopting more efficient management practices, such as crop-livestock integration**,** no-till farming, the use of cover crops, meteorologically informed fertilizer application, precision agriculture, and split (fractional) fertilizer application, supports balanced fertilization and improved soil moisture management. These strategies enhance nitrogen uptake by plants, reducing its conversion to N₂O and subsequent atmospheric emissions. By minimizing excess nitrogen in the soil, they also lower fertilizer input costs, improve soil-plant interactions, and contribute to potential productivity gains and reduced nutrient depletion (Dawson & Hilton, 2011).

###  N_2_O emissions from cultivated pastures: impacts of fertilizers and agricultural techniques

Brazil is the second-largest beef producer in the world, having produced approximately 10 million tons in 2020, equivalent to 14.8% of global market share (FAO, 2021). The country also holds one of the largest dairy markets globally. In 2019, Brazil’s Gross Domestic Product (GDP) totaled R$7.3 trillion, with agribusiness contributing R$1.5 trillion, or 21% of the national GDP. Within this sector, beef cattle production accounted for R$618.5 billion, representing 39% of Brazil’s agribusiness (ABIEC, 2021).

Reflecting the scale of the livestock sector, pastures are the predominant land use in Brazil, covering approximately 154 million hectares, primarily for beef cattle production. However, a significant portion of these pastures, around 52%, exhibits some degree of degradation and supports low animal carrying capacity (MapBiomas, 2024). This situation is at odds with modern global development models, which aim to integrate productivity with environmental sustainability.

Notwithstanding the widespread use of fertilizers on pastures in Brazil, some farms have not yet implemented this practice or use it sparingly, both in quantity and frequency, often limiting their use to the excreta (feces and urine) generated by the animals grazing there. It should be noted that this study did not specify management practices with potential degradation. Furthermore, pastures managed with low environmental conservation practices tend to be more susceptible to soil degradation processes, such as creep and ravine formation, and generally adopt fewer conservation practices, such as crop rotation and productive integration—strategies more common on medium- and small-sized farms.

This type of pasture is prone to erosion; nevertheless, due to the inherently low fertility of tropical soils, the effects on N₂O emissions remain insufficiently understood. Further research is necessary, particularly in relation to fertilized and well-managed pastures.

According to surveys presented by MapBiomas (2024), between 2000 and 2020, 28 million hectares of pastureland went from severe and intermediate degradation to no degradation. On the other hand, Skorupa and Manzatto (2019) and Manzatto et al. (2020) estimated that, by the 2020 s, 16.2 million hectares were occupied by integrated crop-livestock-forestry systems. The reduction in degraded pasture areas, combined with the increase in integrated areas, is indicative of livestock intensification, especially when considering the growth of the national herd and productivity per area. Productivity has risen due to genetic improvements, better soil management, proper fertilization, and environmentally informed practices.

According to the ILPF Network (2021), national trends show increased integration, rotational systems, and fertilizer use, which in turn intensify livestock impacts on nitrous oxide emissions, especially when factoring in the variable “occupied land use area”. This pattern is consistent with the higher EFs observed in pasture systems receiving mineral fertilization and excreta inputs, particularly in the Cerrado and Atlantic Forest biomes and under Cwa climatic conditions (Supplementary Table S1).

Pasture intensification drives livestock farming development in Brazil through fertilization, rotational grazing, legume intercropping, pasture renovation, and integrated crop-livestock systems. Understanding how these practices affect GHG emissions, especially N₂O, is essential. Additionally, higher animal density from intensification increases excreta, impacting N₂O emissions in these systems.

Pastures are perennial agricultural systems that require careful management of both soil fertility and livestock to ensure long-term productivity. They can undergo periodic maintenance or complete renewal. Maintenance involves no soil disturbance and typically includes controlling invasive plants, correcting soil acidity, and applying fertilizers. Renewal, on the other hand, entails desiccating the existing forage, preparing the soil, correcting acidity, sowing new pasture, and applying fertilizers, either prior to planting or as topdressing (Ayer et al., 2015; Bertol et al., 2019; Duku et al., 2025; Ghosh et al., 2024; Pophiwa et al., 2025; Pulido-Moncada et al., 2022; Ritchie & Roser, 2021). In the present review, the highest EFs were observed in clayey Ferralsols (Latosols), under Cwa climatic conditions, reinforcing the importance of considering soil and climate interactions when designing mitigation strategies.

Collectively, the analyses of sugarcane, grain, and pasture systems reveal consistent patterns in N₂O emissions driven by fertilization practices, soil properties, and climatic conditions. These findings support the identification of mitigation strategies, such as improved nitrogen use efficiency and integrated production systems, while also highlighting key data gaps, uncertainties, and regional limitations that must be addressed to improve emission estimates and guide future mitigation efforts.

### Practices and management to reduce N_2_O emissions in agriculture

Kim et al. (2013) investigated the relationship between N₂O emissions and fertilizer application rates and found that direct N₂O emission factors may remain constant, increase, or decrease nonlinearly with changes in N input. Their analysis showed that emissions rise sharply when N application exceeds plant uptake capacity. The surplus N not only serves as a source for additional N₂O production but can also indirectly promote emissions by inhibiting the biochemical reduction of N₂O, underscoring the importance of careful fertilizer management in mitigating emissions.

In temperate regions, agricultural N₂O emissions generally range from 0.5 to 1.5 kg N₂O-N ha⁻^1^ year⁻^1^, largely due to cooler temperatures and reduced soil microbial activity. In contrast, emissions in tropical regions can exceed 3 kg N₂O-N ha⁻^1^ year⁻^1^, driven by higher temperatures and humidity that accelerate nitrification and denitrification processes. For instance, studies indicate that tropical agricultural systems in Brazil emit substantially more N₂O than temperate systems in Europe (Ghosh et al., 2024; Ritchie & Roser, 2021; Skiba et al., 1997). However, in the present review, higher emission factors were frequently associated with lower temperatures, indicating that interactions among climate, soil properties, and management practices may exert a stronger influence on N₂O emissions than temperature alone.

Organic waste has become increasingly important in agriculture. Materials such as straw (crop residue), bagasse (a by-product of processing), and effluents like vinasse are widely used for multiple purposes, from soil cover to prevent erosion, to serve as sources of nutrients and organic matter. These materials help regulate water retention and soil aeration, promoting plant growth. Additionally, they can be used as an energy source and, in some cases, as animal feed (Sherwood, 2020).

These management practices, when combined with mitigation strategies that integrate vegetative, soil, mechanical, and digital conservation measures, can substantially reduce surface runoff and, consequently, agricultural GHG emissions. The integration of intercropping and holistic management practices with mechanical techniques, such as pasture rotation, infiltration basins, no-till farming, contour lines, and terracing, combined with precision agriculture, significantly improves the efficiency and resilience of production systems. These approaches are designed to optimize nitrogen fertilizer application, enhance irrigation efficiency, and enable precise climate monitoring. As a result, they foster sustainable and efficient agricultural management by increasing productivity, minimizing environmental impacts, conserving natural resources, and contributing to improved quality of life (Ayer et al., 2015; Bertol et al., 2019; Duku et al., 2025; Ghosh et al., 2024; Pophiwa et al., 2025; Pulido-Moncada et al., 2022; Ritchie & Roser, 2021). This interpretation is supported by the lower EFs observed in integrated crop-livestock systems compared with conventional grain production systems in the present review.

In addition, it is important to highlight the strategic role of financial instruments, such as green credit lines, conditional subsidies, and payments for ecosystem services, in promoting sustainable agricultural practices. In Brazil, this approach is supported by the National Policy on Payment for Environmental Services (Law No. 14,119/2021) and by emerging initiatives in voluntary carbon markets, aligned with international frameworks such as the Paris Agreement and ESG-driven mechanisms. In this context, RenovaBio (Brazil, 2017) plays a central role by establishing a market-based mechanism that certifies the carbon intensity of biofuels and issues decarbonization credits (CBIOs), thereby incentivizing producers to adopt more efficient and lower-emission practices while contributing to national climate targets.

### Limitations and implications for management and future research

The database used presents several limitations related to the spatial distribution of the reviewed studies (Fig. 1), with a strong concentration of observations in biomes such as the Atlantic Forest, Cerrado, and Pampa, while regions like the Amazon are underrepresented, and no studies were identified for the Caatinga and Pantanal. A similar pattern is observed across climatic zones, where several climate types remain unrepresented. Additionally, due to prevailing agricultural conditions, there is a predominance of Latosols, limiting comparisons with other soil classes. Furthermore, only a limited range of soil management practices and agricultural systems is represented. This should also be interpreted considering temporal variability, as differences across seasons (e.g., wet and dry periods) may significantly influence N₂O emissions and contribute to the observed variability.

These findings support the adoption of Tier II/III approaches over default Tier I factors, improving accuracy and representativeness, while enabling evaluation of management practices. They also highlight the need to further develop regionally calibrated approaches and, in the future, advance toward Tier III methodologies to better capture agroecosystem heterogeneity and enhance life-cycle assessments and mitigation strategies. However, despite advances over IPCC (2019) default values, significant gaps in environmental data remain across both tropical and temperate regions. The limited explanatory power of the models and the wide confidence intervals of emission factors (EFs) reflect the complex controls on N₂O emissions, suggesting that management practices may be as influential as, or even more influential than, edaphoclimatic conditions, underscoring the need for further studies to enable more robust and reliable assessments.

## Conclusions

i. The Tier II emission factors developed here improve the representation of N₂O emissions in Brazil, supporting RenovaBio as well as broader applications such as GHG inventories and LCA, although their use remains dependent on data quality and availability.
ii. Higher emission factors were generally associated with subtropical grain systems, tropical and subtropical sugarcane systems, and pasture systems under Cwa climatic conditions. Irrigation increased emissions in grain systems, although responses varied among land uses and studies.
iii. Weighted emission factors ranged from 0.14 to 1.63%, with national averages within IPCC uncertainty ranges. Pasture emissions with excreta were slightly higher than the IPCC default but highly variable due to limited and uneven data.
iv. Tier II factors are more representative than Tier I defaults; however, data gaps, sampling heterogeneity, geographic bias, and limited model explanatory power contribute to high variability and uncertainty, requiring caution in extrapolation.
v. Management practices that improve nitrogen use efficiency show mitigation potential, but their effectiveness is strongly context dependent.
vi. Overall, advancing regionally calibrated Tier II factors and, in the future, transitioning toward Tier III approaches is essential to reduce uncertainties and support robust assessments and mitigation strategies.

## Supplementary Information

Below is the link to the electronic supplementary material.

ESM 1 (DOCX 866 KB)

ESM 2 (CSV 4.33 KB)

## Data Availability

The data supporting the findings of this literature review will be made available as *Supplementary Interactive Plot Data (CSV)*.
