# Supplementary material for: Nitrous oxide emission factors from fertilizer use in Brazilian agricultural systems: meta-analytical insights for soil management, climate, and land-use policies
Source: Environ Monit Assess. 2026 Jun 26;198(7):775. doi: 10.1007/s10661-026-15560-2 (PMC13309376; doi:10.1007/s10661-026-15560-2)
Supplement: Supplementary file 1 — (DOCX 866 KB) [file 10661_2026_15560_MOESM1_ESM.docx]

**SUPPLEMENTARY MATERIAL**

1. **Linear relationship between environmental conditions and N2O emission factors in different cropping systems**

***1.1 - Sugarcane***

| 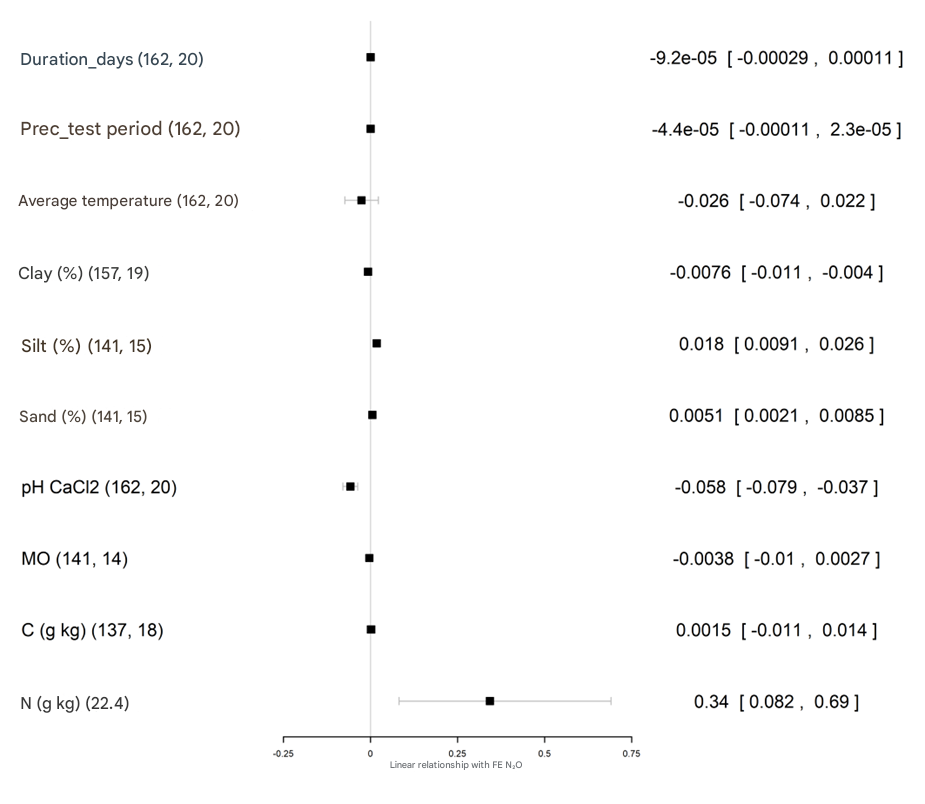  **Figure 1.** Linear relationship between doses of mineral, organic, and mineral+organic fertilizers in sugarcane and N2O emission factors found in the tests. B. Linear relationship between quantitative factors such as test duration (days), precipitation during the test period, average temperature, clay (%), sand (%), silt (%), pH, M.O., C, N, and N_2_O emission factors observed in the test.  **1.2 - GRAINS** |
| --- |

***
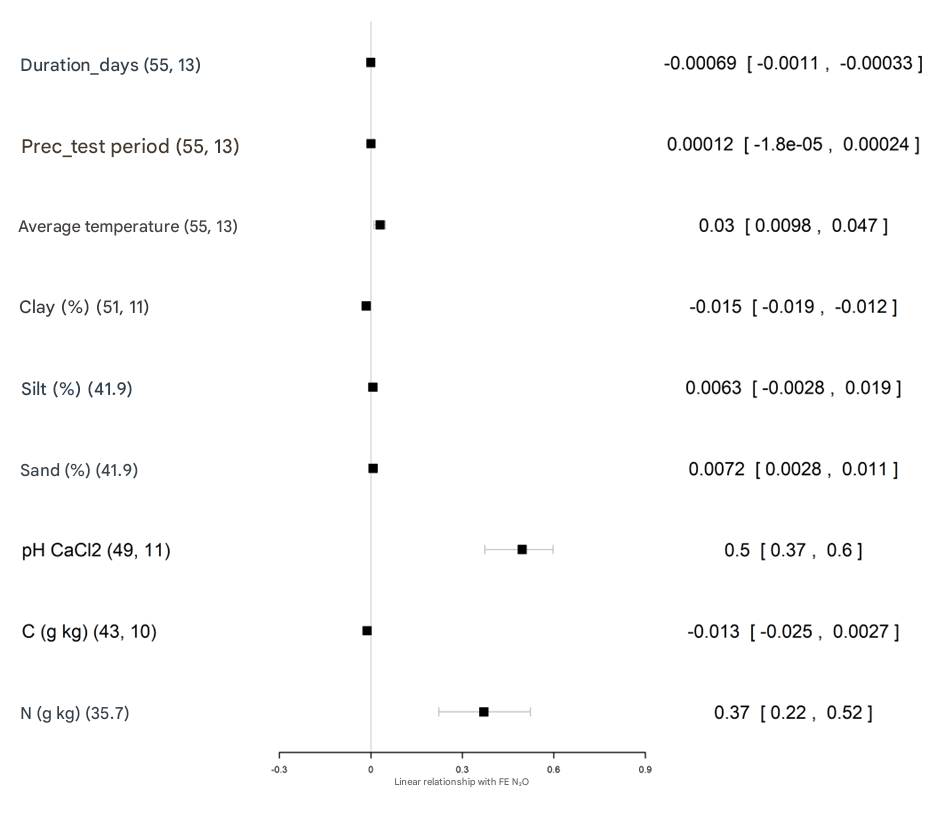
***

**Figure 2.** Linear relationship between mineral, organic, and mineral+organic fertilizer doses in pastures and N_2_O emission factors found in the tests. B. Linear relationship between quantitative factors such as test duration (days), precipitation during the test period, average temperature, clay (%), sand (%), silt (%), pH, M.O., C, and N, and N_2_O emission factors found in the test.

**1.3 - PASTURE – EMISSION FACTOR FROM TESTS WITH MINERAL FERTILIZERS**

**
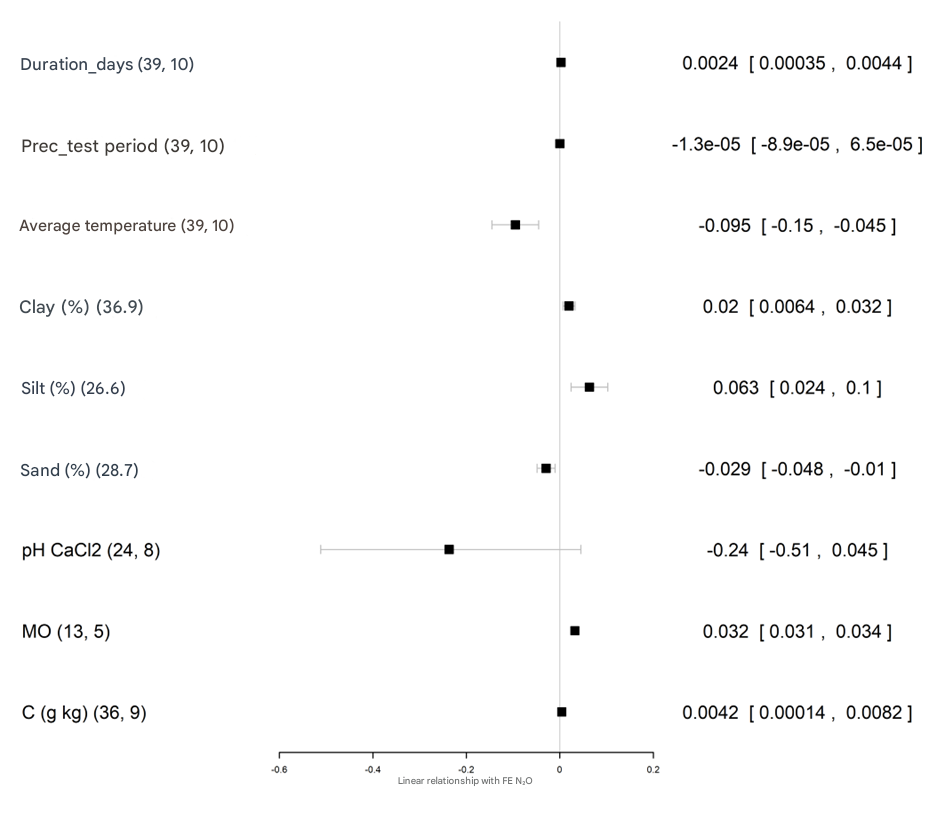
**

|  |
| --- |

**Figure 3.** Linear relationship between quantitative factors such as test duration (days), precipitation during the test period, average temperature, clay (%), sand (%), silt (%), pH, M.O. and C and N2O emission factors found in the test

- 1. **- PASTURE – EMISSION FACTOR FROM EXCRETA**

**
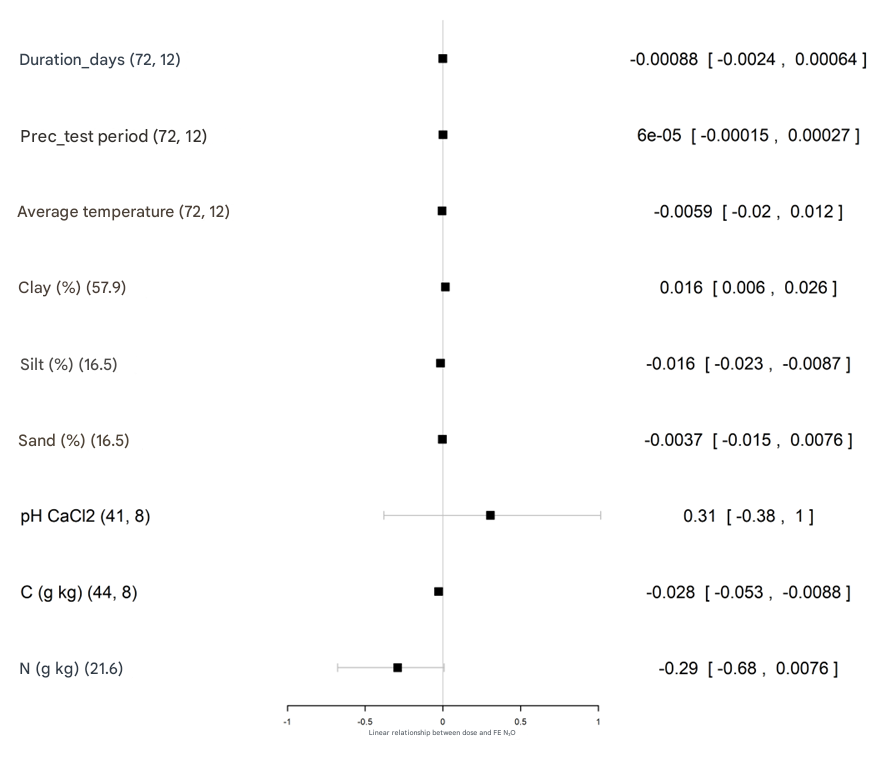
**

|  |
| --- |

**Figure 4.** Linear relationship between quantitative factors such as test duration (days), precipitation during the test period, average temperature, clay (%), sand (%), silt (%), pH, M.O. and C and N2O emission factors found in the test

**2 –** **Linear regression to evaluate the effect of air temperature, precipitation, soil physical properties, pH, MO, C and N on N_2_O emission factors in pasture, grain and sugarcane systems**.

**
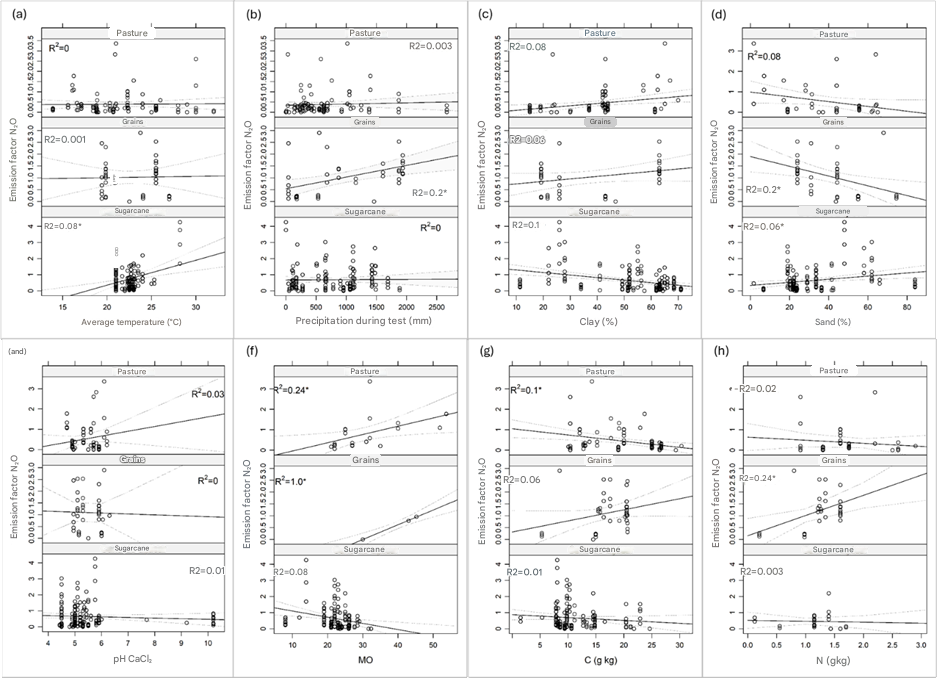
**

**Figure 5.** Linear regression between (A) mean daily temperature, (B) precipitation during the experiment, (C) clay (%), (D) sand (%), (E) pH, (F) OM, (G) soil carbon, and (H) soil nitrogen and N_2_O emission factors in pasture, grain, and sugarcane systems.

***3.4 - EVALUATION OF FERTILIZER DOSES***


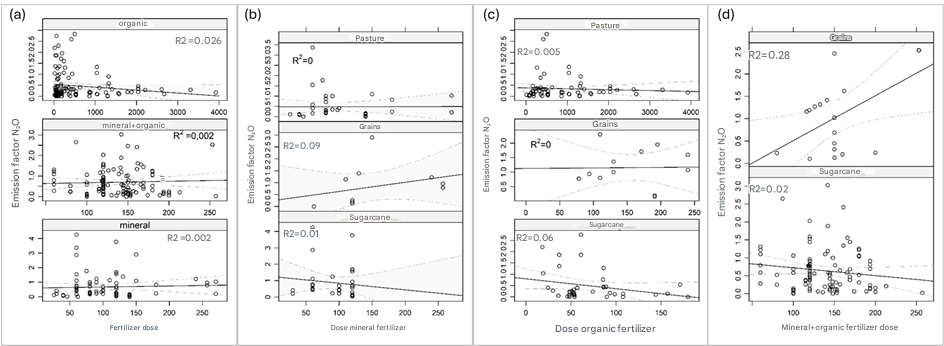


**Figure 6.** Linear regression between total fertilizer dose, mineral fertilizer, organic fertilizer and mineral+organic fertilizer and N_2_O emission factors in pasture, grain and sugarcane systems.

**Table S1.** Mean N₂O emission factors (EFs), standard deviation (SD), and Kruskal–Wallis grouping results for pasture, grain, and sugarcane systems under different climate classes, biomes, irrigation conditions, soil classifications, soil textures, cultivation practices, and production systems in Brazilian agricultural systems.
